# Intermittent Subcutaneous Injections of Apomorphine in Parkinson’s Disease

**DOI:** 10.5152/eurasianjmed.2022.22134

**Published:** 2022-12-01

**Authors:** Mustafa Ceylan, Murat Gültekin, Nazlı Durmaz Çelik, Bedia Samanci, Gül Yalçin Çakmakli, Rezzak Yilmaz

**Affiliations:** 1 Department of Neurology, Atatürk University Faculty of Medicine, Movement Disorders and Neuromodulation Center, Erzurum, Turkey; 2 Department of Neurology, Erciyes University Faculty of Medicine, Kayseri, Turkey; 3 Department of Neurology, Osmangazi University Faculty of Medicine, Eskişehir, Turkey; 4 Department of Neurology, İstanbul University İstanbul Faculty of Medicine, Behavioral Neurology and Movement Disorders Unit, İstanbul, Turkey; 5 Department of Neurology, Hacettepe University Faculty of Medicine, Ankara, Turkey; 6 Department of Neurology, Ankara University Faculty of Medicine, Ankara, Turkey

**Keywords:** Parkinson’s disease, apomorphine, motor symptoms, non-motor symptoms

## Abstract

The intermittent subcutaneous injection of apomorphine is highly effective in the management of motor and non-motor symptoms of Parkinson’s disease. Although it has been shown that apomorphine injection can be safely used in selected cases at all stages of the disease, there is no consensus regarding intermittent administration strategies. This review aimed to discuss the indications for intermittent subcutaneous apomorphine use in clinical practice, possible side effects and their management, and contraindicated cases in light of the literature and to present practical recommendations for clinical practice.

**Main Points**

- Intermittent subcutaneous injection of apomorphine is very effective in controlling the motor and non-motor symptoms of Parkinson's disease.
- It can be used in all stages of the disease in suitable patients.
- The resulting side effects are mild and tolerable.

## Introduction

Apomorphine is a dopaminergic molecule belonging to the group of non-ergot dopamine agonists, and it is as effective as levodopa. It has a lipophilic structure; therefore, its bioavailability is low, but this feature also means that it is well absorbed in subcutaneous administration and shows its effects quickly.^1^ Apomorphine is a potent, broad-spectrum dopamine agonist that activates all dopamine receptors (D1-5).^2^ In this respect, its efficacy differs from other dopamine agonists. Apomorphine has been used for centuries as an emetic, sedative, anticonvulsant, and antipsychotic, as well as for the treatment of alcohol addiction and sexual dysfunction.^3^

Although the efficacy of apomorphine on Parkinson’s disease (PD) symptoms has been known for many years,^4^ studies on its use in the treatment of PD were first undertaken in the late 1980s. In one of the first studies evaluating the efficacy of intermittent apomorphine, it was shown to be highly effective in relieving motor symptoms and providing a significant improvement in quality of life.^5^ After many studies over the following years, subcutaneous apomorphine injection was approved for its use in PD by the US Food and Drug Administration in 2004.^6^ In general, it is known that the anti-Parkinsonian efficacy of oral dopamine agonists is lower than that of levodopa due to limited dopaminergic receptor effects. However, unlike other agents, apomorphine’s antiparkinsonian activity is considered similar to levodopa due to its broad dopaminergic receptor effect.^7^ Among the important pharmacokinetic and pharmacodynamic properties of apomorphine are having a similar symptomatic efficacy to levodopa and being the fastest molecule.

The subcutaneous use and certain side effects of apomorphine, which differs from other dopamine agonists with its aforementioned properties and is similar to levodopa in terms of efficacy, raise various questions among patients and their relatives, and sometimes even in physicians. Recently, guidelines on the use of apomorphine have been published by movement disorder specialists.^8^ The current review aims to present information from daily practice concerning cases in which intermittent subcutaneous apomorphine injection is preferred, side effects and coping methods, and contraindications.

## Apomorphine Administration Methods

Apomorphine is used in the treatment of PD in 2 ways: subcutaneous intermittent bolus injection and subcutaneous infusion therapy. The efficacy of subcutaneous apomorphine treatment has been demonstrated in randomized controlled studies.^9,10^ Intermittent administration may be preferred in the management of motor symptoms, motor complications, and non-motor symptoms (Table 1).

The efficacy of intermittent subcutaneous apomorphine injections has been reported to be 99.5% in PD cases with motor fluctuations. In a previous study, this treatment was found to reduce the Movement Disorder Society-Unified Parkinson’s Disease Rating Scale (MDS-UPDRS) motor scores by 62% and well tolerated.^9^ In another study, a 59% decrease was found in the MDS-UPDRS motor scores 20 minutes after the injection, and the efficacy was reduced. The authors also determined that the effect of apomorphine started within an average of 7.5 minutes, did not lead to any problems over a 3-month follow-up, and was effective in the acute treatment of patients during “off” periods.^10^ In another study, therapeutic doses in the “off” periods were reported as 2.7-5.4 mg. It was observed that the symptomatic efficacy evaluated at the 90th minute of the injection was statistically significantly superior to the placebo, and optimal efficacy was achieved at doses up to 6 mg, while there was no significant increase in efficacy at higher doses but increased side effects were seen. As a result, it was suggested that apomorphine could be well tolerated up to the dose of 6 mg.^11^ As seen in all these studies, a rapid and significant improvement in motor symptoms can be achieved with intermittent subcutaneous injections of apomorphine.

The intermittent subcutaneous injection of apomorphine can be lifesaving in some strategic problems in the treatment of PD. In patients with these problems, intermittent injections are very effective in the presence of dose-related or predictable motor fluctuations (such as nocturnal akinesia, early morning akinesia, and end-dose akinesia) that occur with the fluctuation of the plasma level of levodopa. In patients with painful long-lasting cramps, especially in the legs, which are described as “early morning off” developing secondary to the decreased plasma level of levodopa in the early morning, intermittent injections have been shown to significantly shorten time-to-ON.^12^ In a study evaluating intermittent injections, it was reported that the “off” time was significantly reduced, the daily dose of levodopa could be reduced by 5%, and nocturnal akinesia particularly improved with nighttime injections.^13^

Another area of use of intermittent injections is the delayed levodopa effect (delayed “on”) seen in patients with PD from the middle-advanced stage.^14^ The relationship of levodopa response with meals can be variable, which is partly due to the late and sale absorption of levodopa from the small intestines caused by gastroparesis, i.e., delayed gastric emptying when taken after a meal. This results in levodopa taking up to 1-1.2 to show its effects, and this delayed “on” period not only prolongs the “off” phase but also negatively affects daily life.^14^ A diet rich in protein causes this process to be further prolonged.^15^ Patients are recommended to follow protein-poor diets and take the levodopa preparation on an empty stomach, but this is not well tolerated by some patients. Especially in this patient group, the subcutaneous injection of apomorphine at the time of taking levodopa accelerates the effect of the latter and shortens the “off” period[Table t1-eajm-54-S1-s71].^16^

Knowledge about non-motor symptoms, which affect the quality of life of patients with PD, has increased in recent years. These symptoms can manifest before the diagnosis or at any stage of the disease. Non-motor symptoms that negatively affect the activities of daily life can be overlooked in daily practice, or the management of these symptoms can be very difficult. While some non-motor symptoms arise from the disease itself, others such as hallucinations, impulse control disorder, and somnolence may also be related to the pharmacological agents used. Many studies have reported that apomorphine is effective in relieving non-motor symptoms,^16-20^ but there are very few studies reporting on the efficacy of the intermittent injections of this agent.^13^ Although dopamine agonists should be avoided in the presence of psychotic symptoms as a general rule, many studies have reported that apomorphine infusion therapy is safe to use in the presence of psychotic symptoms and can even improve these symptoms.^21,22^ This efficacy of infusion therapy has been attributed to the dose reduction of dopaminergic drugs used and the effect of apomorphine on the 5-HT 2A receptor.^23,24^ The reduction rate of oral dopaminergic drugs used in intermittent injections is not the same as in infusion therapy; however, in the management of psychiatric symptoms that occur with dopaminergic treatments, the discontinuation of the symptom-causing drug and intermittent subcutaneous injections of apomorphine are very effective in the management of both parkinsonian symptoms and psychotic symptoms. A similar application can be undertaken if there are difficulties in optimizing oral dopaminergic therapy. In such selected cases, intermittent injections may be considered as an effective treatment option. In the EuroInf-2 study, in which the effects of device-assisted treatments, such as apomorphine infusion therapy, deep brain stimulation, and levodopa-carbidopa intestinal gel application on non-motor symptoms were evaluated, apomorphine was reported to improve mood, cognition, attention/memory, and perceptual problems.^25^ Similarly, in another study, it was determined that cognitive dysfunction did not increase in PD cases in which subthalamic nucleus deep brain stimulation was contraindicated and it was safely used under subcutaneous apomorphine infusion for a 12-month follow-up.^26^ More interestingly, a study showed that amyloid β accumulation was decreased in patients with PD using apomorphine.^27^ Given that 80% of patients with PD have cognitive impairment during the disease process,^28^ and even 10%-15% of mild cognitive impairment may occur at the beginning of the disease course,^29^ the intermittent use of apomorphine can be safely preferred in patients with mild cognitive impairment. It is considered that this treatment will have positive effects on these patients, although there is still a need for comprehensive studies.

Dysphagia affects the vast majority of patients with PD. It is an important barrier to adequate nutrition and oral drug use and increases the risk of aspiration pneumonia. It is known that PD affects all phases of swallowing through different mechanisms.^30^ The effects of apomorphine use on dysphagia have been evaluated in very few studies. There are studies reporting an improvement in swallowing function^31^ and a decrease in salivary flow with apomorphine infusion.^30^ However, to the best of our knowledge, there is no research evaluating the efficacy of intermittent administration. In a previous study, functional improvement was observed with 6 mg apomorphine injection in patients with PD presenting with chronic constipation.^32^ In another study, it was reported that non-motor symptoms such as bladder and gastrointestinal problems, extremity pain, and hallucinations were improved with intermittent administration of apomorphine.^13^ Sleep disorders are also very common in PD. In studies investigating the efficacy of apomorphine in sleep disorders, patients who frequently received apomorphine infusions were evaluated, and the results indicated that this treatment had positive effects on sleep disorders and restless legs symptoms.^33,34^

In brief, the subcutaneous use of apomorphine, with both infusion and intermittent methods, is effective in relieving motor and many non-motor symptoms. Although its effects on motor symptoms are better known, studies on its long-term efficacy in non-motor symptoms are still limited, and there is a need for further research with larger populations.

### Apomorphine Dose Titration

An apomorphine test should be performed before apomorphine treatment to assess the optimal apomorphine dose and detect possible side effects. To prevent advert reactions during the test, oral domperidone of 10 mg should be used 3 times a day starting 3 days before the test with the last dose of 20 mg should be taken on the morning of the test day. Parkinson's disease treatments should also be stopped 12 hours before the test. Before the procedure, basal motor examination along with electrocardiography and blood pressure measurement should be performed. The test should be started if the patient is eligible and willing to give consent. The test is started with 1-1.5 mg doses. Although there are various applications for the apomorphine test, the effective dose should be determined based on effectivity and advert reaction development which should be checked periodically once 30- or 60-minute intervals during dose increments. Again, 1-2 mg dose increments are made. For each dose increment, the motor examination should be performed, and blood pressure, advert reactions, and pulse should be checked. Unresponsiveness should be considered when no effect was observed at 7 mg and the test should be terminated. In the presence of a partial response, the dose can be increased up to 10 mg.^8,35^

## Side Effects and Their Management

Despite the benefits of subcutaneous apomorphine injection in PD, it is noteworthy that even some of the eligible patients are unable to continue this treatment for various reasons (Table 1). The main side effects and their management are detailed below.

### Injection Site Reactions

Infusion site reactions are the most common side effect in patients receiving apomorphine injections. Although they are more frequently observed as erythema, allergic reactions, and subcutaneous nodules, more severe conditions, for example, necrosis, panniculitis, and abscesses may also be encountered, albeit rare. Hygiene measures are also related to factors such as the depth and duration of the injection. Although nodules are infected and rarely require antibiotic or surgical treatment, the presence of severe nodules may prevent apomorphine absorption.^36^ In order to cope with milder conditions, such as erythema and subcutaneous nodules, which is the reason for the discontinuation of the drug in many cases, changing the injection site each time, paying attention to skin hygiene, massaging the injection site, using special needles, replacing needles for each injection, and focal ultrasound therapy are frequently recommended methods.^36-38^

### Nausea–Vomiting

Another common side effect that can be encountered especially at the beginning of treatment is nausea and vomiting. This peripheral effect reflecting the agonist activity of apomorphine is more common in intermittent administration than in continuous infusion.^39^ In a study, nausea was observed in one-third of the patients and vomiting in approximately one-tenth.^36^ This side effect can be reduced by starting antiemetic drugs, such as domperidone and trimethobenzamide before treatment.^40^ However, in the use of domperidone, since it causes QT prolongation, care should be taken to use the lowest dose for the shortest time possible. Domperidone should be prescribed with caution in all patients, especially those with underlying heart disease, heart rhythm disorder, and electrolyte imbalance after checking their electrocardiogram (ECG) findings. It is generally recommended to start 1 day before and continue for 3 days to 6-8 weeks depending on the patient’s condition.^34^ Since tolerance develops within 1-1.5 months to nausea and vomiting side effects of apomorphine, the need for antiemetic use should be re-evaluated at regular intervals, and the patient and his/her relatives should be informed in detail about this issue.

### Orthostatic Hypotension

Similar to nausea and vomiting, orthostatic hypotension is more common in intermittent administration; however, its incidence is rarer than nausea.^41^ This side effect can be seen less frequently in patients with the long-term use of dopaminergic therapy.^16^ The effect of orthostatic hypotension is dose-dependent, occurring within 20 minutes after administration and may persist for up to 90 minutes. Therefore, it should be used with caution or avoided, especially in patients with a history of orthostatic hypotension, and caution should be exercised when using it with other drugs that may cause this side effect. For example, ondansetron should not be preferred for nausea since it may increase the risk of severe hypotension and syncope. In addition, patients are advised to avoid alcohol consumption because it may potentiate the hypotensive effect of apomorphine. Similarly, if there is ongoing antihypertensive treatment, it should be reviewed. To manage orthostatic hypotension, which is usually seen at the beginning of apomorphine treatment but shows progression, it is recommended to consume sufficient fluids (2-2.5 L/day), consume sufficient salt, use compression stockings above the knee, avoid sudden position changes, and use fludrocortisone and midodrine treatments when necessary.^36^

### Sedation

Among the common central dopaminergic side effects of apomorphine are somnolence, excessive daytime sleepiness, and sedation, especially at the beginning of treatment,^41^ but they usually regress after the first few weeks. In a study, it was shown that somnolence developed in 18% of patients, and it caused the discontinuation of treatment in 2%.^36^ Dizziness and yawning are common but mild side effects are observed at the beginning of treatment. Considering the possibility of these side effects, it is important to inform the patient that he/she should avoid using machines and vehicles that require attention at the early stage of treatment.^36^ It is also important to improve the quality of sleep at night. If these complaints persist during treatment, modafinil can be tried.

### Neuropsychiatric Side Effects

Although neuropsychiatric complications of apomorphine are rarer than observed in other dopamine agonists, they may still occur. Confusion, hallucination, and agitation are the most common neuropsychiatric complaints and are more frequently seen in individuals with a previous history of similar reactions to dopamine agonists. Studies have reported the incidence of these side effects to reach 14%.^36^ In most cases, these complaints are mild and rarely require the discontinuation of treatment. In a study evaluating the long-term efficacy of apomorphine infusion therapy, it was reported that 5% of patients discontinued treatment due to psychosis.^42^ The risk of neuropsychiatric side effects is higher in patients with pre-existing psychosis or cognitive impairment, as well as those taking high doses of apomorphine.^43^ Therefore, apomorphine treatment may not be very suitable for people who have both cognitive complaints and neuropsychiatric conditions, such as Lewy body dementia and PD dementia. However, it can be used in the presence of mild cognitive impairment with a careful cognitive and psychiatric evaluation before and during treatment. Quetiapine and clozapine can be preferred for the treatment of mild neuropsychiatric side effects that occur during treatment.

Although dopamine dysregulation syndrome and impulse control disorder have also been reported as the side effects of apomorphine, they seem to be relatively rare when compared to other dopamine agonists.^44^ The reason why these side effects are less with apomorphine is due to its lower D3/D2 efficiency ratio than other agonists.^40^ Binge eating-snacking, moderate hypersexuality, and gambling addiction have also been described, but they are rarely severe enough to discontinue treatment. It is known that in most cases, the problem disappears with the reduction of the apomorphine dose and the dose reduction or discontinuation of oral dopamine agonists, if used together. Quetiapine and clozapine can be used in cases where symptoms are severe. Informing the patient’s relatives about these possible side effects is important for early awareness and intervention.

### Eosinophilia and Hemolytic Anemia

Eosinophilia can be detected, albeit rare, in the complete blood count analysis, especially in the early phase of treatment, but it often regresses spontaneously. Hemolytic anemia is a rare but serious side effect of apomorphine^45^ and can be seen in up to 6% of patients receiving continuous apomorphine infusion.^17^ Hemolytic anemia often presents with symptoms such as fatigue, difficulty in breathing, dizziness, and cold hands and feet and may be asymptomatic in mild cases. Therefore, basal complete blood count and reticulocyte count analysis and the Coombs test should be performed and repeated every 6 months. Treatment should be terminated when hemolytic anemia is detected, and the patient should be immediately referred to the hematology unit for consultation.

### Arrhythmia

Another very rare but life-threatening side effect of apomorphine therapy is QT prolongation, which especially occurs at high doses (≥6 mg). This possibility can be minimized by taking an ECG before treatment and avoiding the concomitant use of other drugs that may cause QT prolongation. Atrial fibrillation, ventricular bigeminy beat, and even cardiac arrest have also been described, although they are extremely rare.^36^ Since domperidone, which is recommended for nausea and vomiting, is known to have cardiac side effects, the 2 drugs should be used with caution or avoided in patients with a cardiac history or a pathology in the ECG examination.

### Dyskinesia

It is known that dyskinesias increase with the initiation of apomorphine treatment and continue usually for a few weeks. This is more common during intermittent apomorphine therapy, especially in patients with dyskinesia associated with levodopa therapy. In 1 study, the rate of dyskinesia was found to be 24% of patients, and it resulted in the discontinuation of apomorphine treatment in 2%.^35^ However, dyskinesias decrease during the continuation of the treatment, and therefore, it is important not to discontinue the drug, reduce oral anti-parkinsonian treatments if necessary, and inform the patient about this at the beginning of treatment.

### Other Side Effects

Congestive heart failure, other cardiac arrhythmias, musculoskeletal pain, peripheral edema, respiratory distress, falls, confusion, impaired consciousness, dysarthria, lethargy, mood disorder, and personality changes are also very rare side effects associated with the use of apomorphine, which has been mostly reported on a case-by-case basis.

### Contraindications

Pregnancy, history of hemolytic anemia, active psychosis, resistant and severe orthostatic dysregulation, and dementia are listed as absolute contraindications to the use of apomorphine. Relative contraindications include the lack of family or social support, mild-to-moderate hallucination, marked daytime sleepiness, and needle phobia.

## Conclusion

Side effects of apomorphine, which are the most feared factors in the use of this drug with subcutaneous infusion and intermittent injection, are mostly mild and tolerable, and the general approach is to continue the treatment. Since most side effects are mild, transient, or treatable, this process should be managed in patient–patient, relative–nurse–physician collaboration to make it easier for the patients to continue treatment and prevent its discontinuation in those who will benefit from it.

In addition to side effects, the inability of the patient to self-administer subcutaneous injections during the “off” periods may be the reason for the discontinuation of treatment in intermittent apomorphine therapy. Pre-filled pen-injector forms of apomorphine can help solve this problem. The rate of treatment discontinuation can be reduced with the physician being aware that apomorphine is not particularly difficult to administer as intermittent subcutaneous injections and knowing associated side effects and how to manage them during treatment, the patient and his/her relatives being well informed, and the patient being in communication and cooperation with the nurse and physician. Appropriate counseling, support, and monitoring are, therefore, essential components of the management of patients receiving apomorphine.

In conclusion, it should be kept in mind that similar to infusion therapy, intermittent subcutaneous injections of apomorphine are effective in relieving both motor and non-motor symptoms, can be easily used in all stages of the disease, and should be considered as an option in selected patients.

## Figures and Tables

**Table 1. t1-eajm-54-S1-s71:** Indications, Side Effects, and Contraindications of the Intermittent Subcutaneous Administration of Apomorphine in Parkinson’s Disease

Indications	Unpredictable motor fluctuations despite optimal oral therapy	Predictable dose-dependent motor fluctuations	* Nocturnal akinesia*	* Early morning akinesia/dystonia*	* End-of-dose akinesia*	Difficulty adjusting optimal oral therapy due to patient- or dopaminergic drug-related factors	Non-motor symptoms (urinary, gastrointestinal or psychiatric problems, mild cognitive impairment, and sleep-related disorders)	During the surgical waiting period in cases planned to undergo deep brain stimulation	**Side effects**	Injection site reactions (erythema, allergic reactions, and subcutaneous nodules)	Nausea-vomiting	Orthostatic hypotension	Sedation	Neuropsychiatric symptoms	Dyskinesia	Arrhythmia	**Contraindications**	Pregnancy	Hemolytic anemia	Active psychosis	Advanced dementia	Severe orthostatic hypotension	Long QT syndrome
